# Perceptions of Existing School Fruit and Vegetable Marketing Materials Targeted to School Students by School Nutrition Professionals and Youth

**DOI:** 10.3390/nu15092040

**Published:** 2023-04-23

**Authors:** Meg Bruening, Marc A. Adams

**Affiliations:** 1 Department of Nutritional Sciences, College of Health and Human Development, The Pennsylvania State University, University Park, PA 16801, USA; 2 College of Health Solutions, Arizona State University, Phoenix, AZ 85004, USA; marc.adams@asu.edu

**Keywords:** school nutrition, nutrition education, nutrition marketing, fruit and vegetable consumption, marketing materials

## Abstract

Little published research explores the perceptions of school nutrition professionals and youth regarding existing school nutrition marketing materials. A two-phased approach was taken to address this gap. In Phase 1, a national convenience sample of US school nutrition professionals (*n* = 1546; 89% female; 83.3% white; mean age 50 ± 10.2 years) evaluated a sample of marketing materials via a web-based survey. Phase 2 involved a sample of youth (*n* = 61; 51% female, 52.5% Hispanic; 98% participation in free/reduced price lunch) living in the Phoenix, Arizona metro area who completed interviews on the top-rated materials from the school nutrition professionals (Phase 1). Main outcome measures included perceived attractiveness/appeal, comprehension, relevance, motivation/persuasion, and uniqueness of marketing materials. In Phase 1, means/standard deviations of school nutrition professionals’ perceptions across materials are provided. For Phase 2, a thematic/subtheme analysis of the youth interviews is provided. School nutrition professionals found the materials easy to understand (82%); however, they rated the materials low in motivational appeal (48%) and low in their ability to influence the selection or consumption of fruits/vegetables (95%). Youth participants discussed their likes, dislikes, comprehension, social aspects, and health aspects of the materials. Results indicate that existing marketing materials were not highly rated by professionals or youth. Greater investment and research are needed to test and develop nutrition marketing materials for schools, with a focus on enhancing their appeal and impact on fruit/vegetable consumption.

## 1. Introduction

Fruits and vegetable (FV) consumption has been linked to a lower risk of chronic diseases such as cardiovascular disease, cancer, and type II diabetes. Despite the known benefits of FVs, US youth consumption of FVs are far below recommended levels [1,2,3], thus putting the population at an increased risk for developing chronic health conditions across their lifespan. Amid an obesity epidemic, where 22.8% of children in the US between the ages of 6 and 11 and 25.6% of adolescents are obese [2], it is imperative to investigate strategies to encourage FV consumption, a health behavior linked to weight status [4]. Unfortunately, reports suggest that 5.6–7.2% of high school youth do not eat any fruits and vegetables in a given week [2]. In addition to increased risk for health complications, FV intake may also affect school-aged children’s cognitive development and academic performance [5]. A review of the association between dietary intake and academic achievement reports moderate correlations between the healthful dietary intake in youth (lower intake of energy-dense and nutrient-poor foods, higher FV intake) and standardized academic achievement reports [6].

Significant federal investment has occurred to support FV selection and consumption among youth, particularly in schools. However, FV marketing dollars are minimal when this investment is compared to competing food marketing (e.g., junk food marketing). A large-scale investigation of food marketing expenditures aimed at youth found that despite regulatory efforts, 1.8 billion US dollars (USD) was spent among 49 corporations to directly market food and beverages to children between 2006–2009 [7]. In comparison, 57.5 million USD was spent on all federal child nutrition programs in the US (National School Lunch, School Breakfast, Summer Food Service, and Special Milk) combined within the same period (2006–2009) [8,9]. While it is well understood that mainstream food marketing effectively influences the dietary choices of young children and adolescents [10], an assessment of the impact and perception of FV marketing materials by school nutrition professionals and youth is lacking.

School nutrition programs continue to promote FVs in various ways, given their importance to a healthy diet. Assisted by the passage of the Healthy Hunger-Free Kids Act in the US [11], youth participating in the National School Lunch Program (NSLP) are now required to take more FVs, and schools are required to have more variety of FVs, thus providing more choices to the students. In addition, several school-level intervention programs work to enhance exposure to FVs within and outside of the cafeteria to increase consumption over time, such as the Fresh Fruit and Vegetable Program (FFVP). Many federal, state, and foundation-funded programs create and distribute FV marketing materials such as school announcements, table tents, and posters. Team Nutrition is funded by the US Department of Agriculture (USDA) and supports nutrition education in schools, which often focuses on increasing FV consumption [12]. Nutrition messaging via FV marketing/promotional materials may combat the existing tendencies of children to select unhealthy choices despite the increased presence of fruits and vegetables, but an evaluation of the effectiveness of such materials is limited. Given tight school budgets and the fact that most materials were created with federal funds, these mostly unevaluated materials are widely distributed and available free or at low cost to schools.

School nutrition professionals play a vital role in delivering valid nutrition recommendations and resources to students, staff, and parents. Collective insight into the perceived effectiveness of existing FV marketing materials may improve the quality of the content and foster collaboration among nutrition educators and marketing professionals. While internal evaluation of these materials may exist [13], no study has systematically evaluated existing FV promotional materials. Further, there is no literature currently that examines children’s opinions of school nutrition marketing materials, meaning that the content of marketing materials in schools may not be recognized, resonate, or be favored by children and adolescents, which could impact the ability of the materials to influence FV selection and consumption. The purpose of the current study was to examine perceptions of existing FV marketing materials for schools across two phases. Phase 1 sought to explore school nutrition professionals’ perceptions of freely available FV promotional materials. Then, Phase 2 determined the acceptability of existing fruit and vegetable marketing materials among elementary, middle, and high school children to determine what FV marketing techniques and themes students preferred. Results from this study can help inform future school lunch marketing materials and help school professionals choose marketing materials that align with students’ preferences.

## 2. Materials and Methods

This observational study was conducted in two phases. First, we surveyed a US national convenience sample of school nutrition professionals on their perceptions of existing nutrition marketing materials from various sources. Then, taking the professionals’ most highly rated materials from that study, we interviewed youth in the metropolitan area of Phoenix, Arizona, about selected materials. The Arizona State University Institutional Review Board approved all protocols.

### 2.1. Phase 1

#### 2.1.1. Phase 1 Design and Sample

The authors obtained a total of 30 marketing campaigns promoting fruit and vegetables by convenience sample. Campaigns included various-sized posters, table tents, and announcements. Study staff downloaded all freely available marketing materials from these campaigns promoting nutrition in schools, including: Team Nutrition, Team FNV, SNAP, the National Dairy Council, Iowa Department of Public of Health, and the American Heart Association among others. A proportional subset of marketing items was randomly selected from each campaign (with at least one selected from smaller campaigns) to provide a representative sample of 59 marketing materials from all the materials downloaded.

School nutrition professionals were then surveyed on their perceptions of these selected existing and free promotional marketing materials on fruits and vegetables to children in schools. We emailed each state’s NSLP coordinator up to two times for contact information for school nutrition professionals in their respective state. In the case where there was no response from the state NSLP administrator, we searched each respective state’s Department of Education and Department of Health websites for publicly available lists of nutrition professionals. We obtained contact information for school nutrition professionals from 42 states. With this contact information, we sent emails with individual links for a web-based survey to 6317 school nutrition professionals along with two reminder emails to complete the survey in May 2018. A total of 1545 professionals completed the survey within two weeks, after which we closed the assessment.

Each professional was asked to rate three promotional items sequentially. To minimize response bias for the materials, we programmed the web-based survey to randomly select and display 3 promotional materials from the set of 59 to each participant. We estimated that it would require approximately 35 independent professional evaluations to produce a stable mean value for each marketing item. Once each piece reached the threshold of 35 responses, we examined the standard deviations for the responses for the 5 promotional materials with the highest and lowest review scores. As the standard deviations were similar for these promotional materials, we closed the survey. Upon completing the survey, participants were provided with a USD 5 gift card and entered into a raffle for USD 100 and USD 50 gift cards.

#### 2.1.2. Phase 1 Measures

Survey measures for the school nutrition professionals were developed for this study (see Table 1). The development of the questions was guided by the pretesting constructs from USAID [14], including: purpose, attractiveness/appeal, comprehension, relevance, motivation/persuasion, and uniqueness. School nutrition experts, including a local food service director and leaders from the Arizona Department of Education, examined the survey for face validity. It was further pretested with local school nutrition professionals (see Table 1 for measures).

### 2.2. Phase 2

#### 2.2.1. Phase 2 Design and Sample

In this phase, a sample of youth participants (21 elementary students, 20 middle school students, and 20 high school students, balanced by sex) from the Phoenix, Arizona metro area were recruited to participate in an interview on their opinions of the previously selected school fruit and vegetable marketing materials. Parents provided written consent; youth provided oral assent to participate in the study. All marketing materials for this study were chosen from Phase 1 with nutrition professionals. In Phase 2, the top 75% (*n* = 35) of marketing materials from Study 1 were shown to youth. Recruitment and data collection took place from October to December of 2018 and participants received a USD 10 gift card for their participation, which took approximately 30 min.

Youth participants completed an audio-recorded one-on-one interview with trained research staff. At the beginning of the interview, participants were asked to complete a short demographic survey with questions on race/ethnicity, age, sex, and grade level. During the interview, participants were asked to rank 35 different school marketing materials from most favorite to least favorite in four categories: table tents (*n* = 10), medium posters (*n* = 10), large posters (*n* = 7), and announcements (*n* = 8). Again, all marketing materials were selected from free, online resources to increase the generalizability of findings. Marketing materials were shown in a randomized order to minimize any order effects on rankings.

#### 2.2.2. Phase 2 Measures

After completing the demographic survey and ranking marketing materials, youth were asked a series of open-ended questions on their favorite marketing material in each of the four categories. Questions were only asked about their favorite items for the sake of time. Using the Johns Hopkins Social Marketing Guide constructs (Table 1), similar to the school nutrition professionals, questions were asked determining purpose, attractiveness/appeal, comprehension, acceptance, relevance, motivation/persuasion, and uniqueness.

### 2.3. Phase 1 and 2 Analyses

For Phase 1, descriptive analyses (mean and started deviations) were calculated for professionals’ rating of the materials, perceptions of influence of promotional material, and likably of the materials. For Phase 2, research assistants transcribed audio interviews to text. After the interviews were completed, a codebook was created for the themes and the transcriptions were analyzed and coded using NVivo software by S.P. and K.K. Any discrepancies in coding were discussed between raters and if there was no consensus, M.B. made the final decision.

## 3. Results

### 3.1. Phase 1 Demographics

Respondents to the survey included school nutrition managers (typically those who oversee a school nutrition program in an individual school), school food service directors (typically those that oversee school nutrition programs in multiple schools), state agency officials who oversee state-wide school nutrition programs, and school nutrition advocates (those who advocate for healthier school meals in the US), among others. The majority of respondents were female (88.7%), non-Hispanic (89.5%), and white (83.3%). Just under 10% were registered dietitians (Table 2). On average, respondents had extensive experience with school nutrition with a mean of 10.0 ± 7.9 years in their current position and with a mean age = 50.0 ± 10.2 years. Most of the respondents were affiliated with public schools (74.7%), although a significant proportion were also associated with charter schools (10.7%). Most were also affiliated with K-12 schools (59.3%).

### 3.2. Perceptions of the Materials Assessed by School Nutrition Professionals

Across the 64 marketing materials, respondents had difficulty identifying the purpose of marketing items which were to encourage students to take, consume, both, or neither regarding FVs (Table 3). Professionals ranked the majority of materials displayed to them as easy to understand (82.2%); however, most rated the item relevance as relatively low (on a scale of 1–10, the average personal like of each piece was just over 5, and approximately 83% indicated the materials did not highlight foods that children would want to eat). Professionals reported that the 52% of materials displayed to them would motivate children, but only 5% of displayed items would be influential for either taking or eating FVs. Only about half of the respondents indicated that the displayed materials were unique.

Generally, professionals rated the posters highest and announcements ranked the lowest (Table 4). School nutrition professionals reported that they liked the use of humor, color, and common foods used in the marketing materials. Conversely, too much text and the lack of prompts/nudges/reminders/calls to action were viewed as negatives by the respondents.

### 3.3. Phase 2 Participant Demographics

Youth participants were evenly split across elementary, middle, and high school students, as expected by the sampling design. However, one more elementary student participated than the other groups for a total of 61 youth participants (Table 2). The interviews were relatively balanced by sex (51% female). Youth participants were diverse with 58.6% non-white and 52.5% were Hispanic or Latino. Almost all (98%) participated in the National School Lunch Program.

### 3.4. Phase 2 Themes and Subthemes from the Interviews

Among the 61 student interviews, five themes emerged: likes, dislikes, comprehension, social aspects, and health aspects. Twenty subthemes were identified related to these themes. The frequency of these themes and subthemes are presented in Table 5.

The theme of ‘likes’ was defined as the student’s favoritism toward a particular FV and the way it is marketed to them. This theme includes the subthemes of FV item, visual aspects, information, humor, length, and cartoons and media. The most prevalent subtheme under this category was liking the particular FV pictured. One participant discussed their favorite fruit: “I like how it’s about watermelon, that’s my favorite fruit and a lot of them added like sweet, delish vitamin A and C. I think that kind of makes it more centered toward us.”

Many participants noted how the visual aspects such as color and design played a large role in attracting them to marketing materials. One participant stated: “I chose this one for kind of the, like visual aspect of it. And like the graphic design. Because I think it would be also eye catching. I think that’s a very important part. Cause like a good balance between having a lot of information and the, the visual. I like sort of the rainbow.”

The theme of ‘dislikes’ was defined as the student’s issues with the marketing materials. Five subthemes were identified under the dislikes theme including: FV pictured, age appropriateness, visual aspects, length, and font. The most prevalent subtheme under this category was that the youth did not like FVs. One participant stated: “Spinach is disgusting.”

Another popular theme amongst the dislikes was that some students felt that marketing materials were not age appropriate. One student noted: “I actually really like it. But maybe it would be kind of childish for like high schoolers. Maybe, I think, it would really work in elementary school. Like I know if I was in elementary school I would like this.”

The theme of ‘comprehension’ was defined as students’ understanding of advertising messaging and the particular FV pictured/mentioned, and includes the subthemes: did not recognize a particular FV, confusing, and had not tried a particular FV. Participants also reported that they found some marketing materials to be confusing. One participant said she did not understand what the marketing material was telling her: “[the ad is] talking about the food and your digestive system and what digests like faster and what digests slower.” Other participants had trouble recognizing the FV pictured in the marketing materials. One participant said the following about a marketing material that pictures many different vegetables: “I can’t tell if that’s onion or um… I don’t know what it’s called.”

The theme of ‘social influences’ was defined as participant perception of their diet, their peers’ diets, and FV preferences. The two subthemes under social influences included: peers eat unhealthily and peers liking the FV pictured. Peers eat unhealthily was the most prevalent subtheme. When asked what another child of similar age would think of a marketing material, a participant responded: “most of the kids my age think that vegetables are not good, and that they like [unhealthy] food more than vegetables.” Another participant said: “kids are more leaning towards junk foods or foods that are fast food made and they’re not really healthy for you so this message is something that a lot of people need to hear now.”

The theme of ‘health aspects’ was defined as participant recognition of health aspects of FV and its implications. This theme encompasses four subthemes: information about the importance of FV consumption, about vitamins and minerals, information about the future impact of diet and nutrition, and motivation to eat more FVs. Participants discussed the future impact of diet and the implication of their dietary choices. One participant said: “if we don’t start eating healthy at a younger age you can grow up to be unhealthy and it will be harder to go back to being healthy.” Many participants also preferred advertisements that included facts about FVs or information on vitamins and minerals. One participant said: “[peers would] probably like it because they know that like if their eating these [fruits] they’re like getting the right nutrients and minerals for their body.”

## 4. Discussion

Through a two-phase study, we aimed to better understand how school nutrition professionals and youth view free, publicly available nutrition marketing materials to promote healthful eating in school cafeterias. In general, the freely available marketing materials were not perceived very positively, but consistent themes across both populations were identified. Materials that promoted foods that were already well liked amongst students were more highly rated and/or ranked by the nutrition professionals and the youth. Other mentioned likes were materials that were more visually appealing, those which presented clear information, and those which incorporated humor. Most materials were not perceived by professionals or students to motivate consumption of fruits and vegetables. More work is needed to develop and evaluate more impactful nutrition marketing materials to help school nutrition efforts.

Although it is well researched that proper marketing influences children’s and adolescents’ dietary habits, to our knowledge, no research has explored how school nutrition professionals and students perceive healthy school lunch marketing materials. Generally, the materials were described as understandable, appropriate for the target population, familiar, and motivating. The use of humor and visually appealing materials were more highly rated, a finding that is consistently supported by marketing research. Unfortunately, on average, school nutrition professionals rated the existing materials not very highly with an average score of 5 out of 10 for most components. There was also little agreement from the perspective of the school nutrition professionals on the purpose of the materials (e.g., select or consume FVs, or both). Given the general purpose of the marketing campaigns, this seems a particularly important finding. Similarly, some youth described the messages of the top-rated materials as confusing. While these materials are freely available and may provide an appealing aesthetic to the school nutrition environment, the value of these materials to professionals or students for eliciting their desired response is lacking. Based on our findings with the nutrition professionals, results suggest that the materials are motivating, but do not reach a critical threshold for evoking children to take or eat FVs. The materials are not free to create [12], and if they are not effective, perhaps those dollars should be directed toward other efforts. Alternatively, perhaps more investment is needed to ensure that nutrition marketing materials effectively increase selection and/or consumption of FVs.

Both professionals and students indicated that they liked materials that promoted food that were already popular with students. The youth, particularly the elementary students, described being motivated to eat a food if they knew they already liked it. This finding suggests that passive nutrition marketing such as posters and table tents may be more useful in prompting the selection or consumption of known foods, but not be effective at encouraging students to take or to consume new foods. We did not assess how these materials were used in schools. Research is needed to explore the efficacy of passive nutrition marketing when paired with other nutrition strategies. Choice, taste tests, and the use of peer influence have been shown to be effective at helping to promote acceptance of new items [15,16]. The location of the marketing materials may need to be considered in addition to the qualities of the piece. For example, table tents on cafeteria tables prompting students to *select* an FV item may not be the ideal location, as the opportunity to select a food item has already passed. Rather, table tents that promote consumption of FVs already on the plate may be more salient to students and effective at increasing consumption as the opportunity for consumption is more proximal [17,18].

Another subtheme which the youth often reported to impact their perception of the nutrition marketing material was perceived peer opinion. Many youth participants reported that friends eat unhealthily. However, youth participants who noted that their friend group would like the fruit or vegetable shown, were more likely to speak more favorably about the marketing material. Perceived peer influence has been supported by research to suggest that peers can impact both healthy and unhealthy eating behaviors among youth [19,20,21]. Future research should look at how positive peer modeling can have an influence on marketing material preference as well as FV consumption.

Novelty may have played a role in the findings. In the literature, novelty increases interest in marketing materials and FV consumption; however, FV intake often returns to baseline levels once the novelty wears off [15,22]. Moreover, marketing materials are often displayed for long periods. Indeed, some of the materials obtained included popular celebrities (e.g., DJ Khalid) and cartoons from many years or a decade ago, which may be unrecognizable to current students. Future research needs to examine the effect of FV marketing when the marketing materials consider student preferences.

This study has several limitations. First, the surveys and interviews include opinions and perceptions of selected materials, which may not predict effectiveness or consumption habits. Additionally, participants have been shown a subset of marketing materials that may not be representative of what is being shown in school cafeterias. While the marketing materials were taken from online sources, they may not represent all fruit and vegetable advertisements available for schools to use. The marketing material were randomly selected and may not represent the best material from each campaign and therefore, results cannot be generalized to a specific campaign. Lastly, the school nutrition professional study included experts from 42 states and the youth study included a convenience sample from Phoenix, Arizona. Due to this, the study may not be generalized to the overall population throughout the United States; however, the youth study sample was balanced by school level (elementary, middle, and high school), ethnicity, race, and sex.

## 5. Conclusions

School nutrition marketing is often used to promote the consumption of healthy foods such as FVs. However, very little published research has examined school nutrition professional and youth perceptions of these materials. Our mixed methods study suggests that there is significant room for improving freely available nutrition marketing materials from both the school nutrition professional and youth perspective. Perceptions of strengths of well-liked materials included use of popular foods and those that are visually appealing. More investment and research are needed to assess the effectiveness of nutrition marketing materials alone and in conjunction with other school nutrition interventions to promote healthy eating behaviors in schools.

## Figures and Tables

**Table 1:** Questions asked of School Nutrition Professionals and Youth to Examine Perceptions of School Nutrition Marketing Materials.

Construct	Professional Items	Student Interview Items
Purpose	In your view, does this piece promote: Taking fruits and vegetables, Eating fruits and vegetables, Both, Neither, Other What were the main messages of this piece? Prompts/nudges/reminders to take fruits and vegetables, Prompts/nudges/reminders to eat fruits and vegetables, Nutritional education/information to take fruits and vegetables, Nutrition education/information to eat fruits and vegetables, Rewards for taking fruits and vegetables, Rewards for eating fruits and vegetables, and/or Don’t know	What do you think this ad is about? What in the picture makes you think that? What do you think of that message? Does it make you feel any certain way? Are there any other messages that you get from this ad? Do you think these messages are important to kids your age? Why?
Attractiveness/appeal	What do you like/most like about the piece? 19 components that they liked and did not like about the piece (e.g., color, layout, relevance to the target audience of student, messaging, use of humor, use of the type of food, use of prompts/nudges/call to action)	What do you like best about this ad? What do you think other students your age would like best about this ad? What, if anything, do you not like about this ad? Does this ad make you like it [the food] any more or less? What do you think your friends would think of this advertisement?
Comprehension	Please rate how easy this piece is to understand for students (not at all, slightly, somewhat, or very understandable)	What do you think this ad is about? What age of students do you think would like this ad?
Relevance	On a scale of 1–10, how much do you like this piece for use to promote fruits and vegetables in school cafeterias? For what grade level(s) is this piece appropriate for? Elementary, middle, and/or high school? Does this piece include foods that you feel students would like to eat?	What is the name of the fruit or vegetable in this advertisement? What kinds of food does this ad have in it that you like? How much do you like the fruit or vegetable in this advertisement?
Motivation	On a scale of 1–10, how much do you think this piece would influence students to take (serve themselves) fruits and vegetables in school cafeterias (1 being not influential, 10 being extremely influential)? On a scale of 1–10, how much do you think this piece would influence students to eat fruits and vegetables in school cafeterias (1 being not influential, 10 being extremely influential)? On a scale of 1–10, how much do you like this piece for use to promote fruits and vegetables in school cafeterias (1 being not influential, 10 being extremely influential)? Rate how well this piece would motivate kids to eat the fruits and vegetables pictured/mentioned (not at all, slightly, somewhat, or very motivating)	Does this ad motivate you to do anything? If so, what?
Uniqueness	Does this piece remind you of other nutrition-related marketing campaigns you’ve seen before?	How much does this ad remind you of other ads you’ve seen before?

**Table 2:** Demographics of school nutrition professionals (*n* = 1546) and youth (*n* = 61) completing school marketing ratings.

School Nutrition Professionals	*n* = 1546
Years in current position, mean ± SD (*n* = 1546)	10.0 ± 7.9 (range 1–42; median = 7)
Age, mean ± SD (*n* = 1510)	50.0 ± 10.2 (range 22–80; median = 51)
Sex, % (*n*) (*n* = 1526) Female	88.7 (1353)
Race , % (*n*) (*n* = 1515) White Black American Indian Asian Native/Pacific Islander Two or more races Prefer not to answer	83.3 (1262) 6.3 (96) 1.3 (20) 0.6 (9) 0.3 (4) 1.6 (25) 6.5 (99)
Ethnicity, % (*n*) (*n* = 1518) Hispanic	10.5 (159)
RD, % (*n*) (*n* = 1456)	9.1 (133)
Highest education, % (*n*) (*n* = 1530) High school or less Some college/trade/associates College graduate Graduate degree	17.7 (271) 31.8 (487) 32.6 (499) 17.9 (272)
Type of school setting, % (*n*) (*n* = 1492) Public Charter Private Religious Tribal Other	74.7 (1115) 10.7 (159) 6.2 (92) 6.4 (95) 0.9 (13) 1.2 (18)
Grades, % (*n*) (*n* = 1491) K-12 K-8 Elementary Middle High Other	59.3 (885) 16.7 (249) 13.6 (203) 2.8 (42) 5.0 (75) 3.3 (49)
Youth Participants	***n* = 61**
Grade, % (*n*)	
3rd	6 (9.8%)
4th	3 (4.9%)
5th	12 (19.7%)
6th	7 (11.4%)
7th	9 (14.8%)
8th	4 (6.6%)
9th	4 (6.6%)
10th	2 (3.1%)
11th	9 (14.8%)
12th	5 (8.2%)
Age, mean ± SD	12.6 ± 2.9
Sex % (*n*)	
Boy	30 (49%)
Girl	31 (51%)
Race, % (*n*)	
White	25 (41%)
Other	20 (32.3%)
Black or African American	10 (16.4%)
Multiple	4 (6.6%)
American Indian/Alaskan Native	2 (3.3%)
Ethnicity, % (*n*)	
Hispanic or Latino	32 (52.5%)
Not Hispanic or Latino	29 (47.5%)
**Participation in the National School Lunch Program**	
Yes, % (*n*)	60 (98.4%)

**Table 3:** Average scores across the displayed images by school nutrition professional raters ^a^.

Construct	Mean ± Standard Deviation
Purpose (% agree)	
Take food item	12.1% ± 9.4%
Eat food item	20.3% ± 11.0%
Both	45.3% ± 16.5%
Neither	20.1% ± 16.2%
Comprehension (% agree)	82.2% ± 12.7%
Relevance	
Like the marketing piece ^b^	5.68 ± 1.1
Target population (% agree)	
Elementary school	64.3% ± 22.6%
Middle school	70.3% ± 17.9%
High school	59.2% ± 23.8%
Piece features food students want to eat (% agree)	17.4% ± 19.0%
Motivation	
Motivating (% agree)	51.8% ± 15.5%
Influence to take FVs ^b^	5.29 ± 0.8
Influence to eat FVs ^b^	5.17 ± 0.8
Uniqueness (% agree)	52.8% ± 17.9%

^a.^ A minimum of 35 raters were randomly assigned to review each marketing piece. ^b.^ On a scale of 1–10, with 1 being a low rating and 10 being a high rating.

**Table 4:** Summary of the five highest- and lowest-ranked marketing pieces by school nutrition professionals.

Rank	Type of Marketing Piece	Number of Raters	Source ^a^	Influence Students to Eat FVs	Influence Students to Take FVs ^b^	Avg. Likability Score
1	Poster	48	National Watermelon Promotion Board	7.1	7.2	7.5
2	Poster	35	American Heart Association	6.5	6.5	7.6
3	Table tent	52	Connecticut Department of Education	7.0	6.4	7.1
4	Poster	49	University of Texas School of Public Health	6.1	6.2	6.8
5	Table tent	44	Iowa Department of Public Health	6.1	6.2	6.8
60	Table tent	60	Team FNV	4.2	4.2	4.3
61	Poster	56	Team FNV	4.3	4.2	4.1
62	Announcement	53	American Heart Association	5.7	5.6	1.1
63	Table tent	54	Team FNV	3.3	3.0	3.0
64	Announcement	42	Team Nutrition	2.7	2.7	3.5

^a^ A minimum of 35 raters were randomly assigned to review each marketing piece. ^b^ FVs = fruits and vegetables.

**Table 5:** Heat map frequency of theme and subthemes of perceptions of nutrition fruit and vegetable (FV) marketing materials by elementary, middle, and high school students (*n* = 61) ^a^.

	Elementary	Middle	High
**Likes**			
Fruit and/or vegetable pictured	78	70	66
Visual aspects	20	30	40
Information	30	33	30
Humor	10	4	6
Length	1	1	20
Cartoons and media	3	13	14
**Dislikes**			
Fruit and/or vegetable	13	12	16
Age appropriateness	9	1	13
Visual aspects	8	6	7
Length/wordiness	1	5	13
Font	3	1	6
**Comprehension**			
Doesn’t recognize FV	19	13	9
Confusing message	10	4	4
Hasn’t tried FV	3	3	5
**Social Influences**			
Peers eat unhealthily	29	29	25
Peers like FV	26	13	8
**Health Aspects**			
Importance of FV	38	15	9
Vitamins and minerals	20	19	30
Future impact of diet	22	14	11
Motivates to eat more FV	28	5	5

^a^ The heat map was automated by NVivo software. Red indicates low frequency of themes, where darker colors indicate lowest frequency; Yellow indicates moderate frequency of themes with darker colors indicating higher frequency; Green indicates high frequency of themes with darker colors indicating higher frequency.

## Data Availability

Data are available upon request.
